# Composition of Bacterial and Archaeal Communities in an Alkali-Surfactant-Polyacrylamide-Flooded Oil Reservoir and the Responses of Microcosms to Nutrients

**DOI:** 10.3389/fmicb.2019.02197

**Published:** 2019-09-27

**Authors:** Peike Gao, Yu Li, Lijie Tan, Fenfen Guo, Ting Ma

**Affiliations:** ^1^College of Life Sciences, Qufu Normal University, Qufu, China; ^2^The Second Oil Production Plant, PetroChina Daqing Oilfield Limited Company, Daqing, China; ^3^College of Life Sciences, Nankai University, Tianjin, China

**Keywords:** oil reservoir, alkaline-surfactant-polyacrylamide, microbial community, 16S rRNA sequencing, *Halomonas*

## Abstract

The microbial communities in alkali-surfactant-polyacrylamide-flooded (ASP-flooded) oil reservoirs have rarely been investigated compared to those in water-flooded oil reservoirs. Here, the bacterial and archaeal communities in an ASP-flooded reservoir and the adjacent water-flooded block, and responses of the microbial communities in microcosms to nutrients were investigated by 16S rRNA gene sequencing and cultivation. Compared with the water-flooded block, both the bacterial and archaeal communities inhabiting the ASP-flooded block had lower Sobs indices (91:232 and 34:55, respectively), lower Shannon indices (1.296:2.256 and 0.845:1.627, respectively) and higher Simpson indices (0.391:0.248 and 0.678:0.315, respectively). *Halomonas* (58.4–82.1%) and *Anoxynatronum* (14.5–18.2%) predominated in the ASP-flooded production wells, and were less than 0.05% in the bacterial communities of the adjacent water-flooded production wells, which were dominated by *Pseudomonas* and *Thauera*. *Methanobacterium* accounted for 65.0–94.5% of the archaeal communities inhabiting the ASP-flooded production wells, and *Methanosaeta* (36.7–94.5%) dominated the adjacent water-flooded production wells. After nutrients stimulation, the quantity of cultivable microorganisms increased from 10^3^/mL to 10^7^/mL. Community analysis indicated that the relative abundances of some species that belonged to *Halomonas* and *Pseudomonas* obviously increased, yet there were no oil emulsification or dispersion and changes of surface tension of the water-oil mixture. In addition, 6 alkali-tolerating strains showing 98% similarity of 16S rRNA genes with those of *Halomonas alkalicola* and *Halomonas desiderata* and 2 strains with 99% similarity with *Pseudomonas stutzeri* gene were isolated from the nutrients stimulated brines. In summary, this study indicated that *Halomonas*, *Anoxynatronum*, and *Methanobacterium* were dominant populations in the ASP-flooded reservoir, the extreme environment decreased microbial diversity, and restricted microbial growth and metabolisms.

## Introduction

Water-flooding is an efficient secondary oil recovery process with pressurized water being pumped into oil-bearing strata to push oil out of reservoirs, and has been employing worldwide (Lenchi et al., 2013; Cai et al., 2015). However, large-pore paths generally form in oil-bearing strata after long-term water-flooding, leading high water content of produced liquids. At this stage, approximate 30% oil reserves was exploited, leaving 70% untapped oil underground. Then, tertiary oil recovery (enhancing oil recovery, EOR) is undertaken. Among them, polymer-flooding is considered a most successful chemical EOR method, and has achieved large-scale field application (Gao, 2013). This technique generally employs hydrolyzed polyacrylamide to recovery the residual oil underground through sealing mainstream channels and increasing viscosity of the displacing phase (Nasr-El-Din et al., 1991; Goodyear et al., 1995). Alkali-surfactant-polyacrylamide-flooding (ASP-flooding) is another EOR method, and has been used to exploit the residual oil underground in high water-cut and depleted oil reservoirs (Carrero et al., 2007; Shen et al., 2009). This technique can effectively improve the sweep efficiency of displacing phase through generating ultralow interfacial tension of water-oil mixture, improving oil mobility, and sealing mainstream channels. Recently, an alkali-microbe-polymer-flooding (AMP-flooding) method for EOR was developed and tested in laboratory, and reported that the AMP-flooding method had identical displacement properties compared with the conventional ASP-flooding method (Wang et al., 2019). In that case, the microorganisms inhabiting oil reservoirs may play an important role in EOR for ASP-flooded reservoir. But so far, our knowledge about microbial communities in ASP-flooded reservoirs is lacking.

Oil reservoirs, characterized by high hydrophobicity, low water activity, high salinity and pressure, and lack of nitrogen, are extreme environments for microbial life (Cai et al., 2015; Pannekens et al., 2019). Nevertheless, oil reservoirs consist of multiphase medium, such as crude oil, formation water and organic materials, where microorganisms can thrive (Kobayashi et al., 2012). To date, a large amount of microbial populations have been isolated or detected in oil reservoirs worldwide (Lenchi et al., 2013; Al-Sayegh et al., 2015; Gao et al., 2016; Okoro et al., 2016; Su et al., 2018). These microorganisms are versatile: some species can degrade crude oil, some can produce biosurfactants (Liu et al., 2015; Cui et al., 2017; Qi et al., 2018), biopolymers (Bi et al., 2016; Xia et al., 2018; Zhao et al., 2018), or biogases (Dong et al., 2015; Zhu et al., 2015), and have been used to mobilize and recover residual oil from oil-bearing rocks (Youssef et al., 2009; Wang et al., 2014; Yi et al., 2018). In addition, nitrate can be used to reduce formation of hydrogen sulfide produced by sulfate-reducing bacteria (SRB) based on the competitive inhibition of nitrate-reducing bacteria on SRB (Gieg et al., 2011; Gassara et al., 2015). It is also a feasible way to control microbial sulfide production by limiting sulfate dispersal in oil reservoirs (Shen et al., 2018). A previous study also used anaerobic chemolithotrophic nitrate-dependent Fe(II)-oxidizing microorganisms to modify rock porosity, with subsequent alteration and improvement of floodwater sweep to improve oil recovery (Zhu et al., 2013). In order to use these microbial populations to improve oil production, it is essential to investigate the microbial communities inhabiting diverse oil reservoirs.

Unlike the bulk of the deep biosphere, oil reservoirs are subject to extremely anthropogenic perturbation (Vigneron et al., 2017). Among them, drilling and flooding are the main anthropogenic factors in oil production process, leading inevitable influences on subsurface microbial communities because of the introduction of external microorganisms and substances. Compared with water injection, the injected alkali, surfactants, and polyacrylamide in ASP-flooding process will inevitably produce greater influences on subsurface microbial communities, in particular, the strong basicity of the displaced fluid can destroy microbial cytomembrane. Therefore, it is very appealing to investigate the microbial communities inhabiting ASP-flooded oil reservoirs. In this study, bacterial and archaeal communities in an ASP-flooded block and the adjacent water-flooded block, located in Daqing Oil Field, Northeast China, were investigated by 16S rRNA gene sequencing. In addition, nutrients stimulation was performed in microcosms to reveal the EOR potential of the microorganisms inhabiting the ASP-flooded block.

## Materials and Methods

### Sample Collection

**FIGURE 1:**
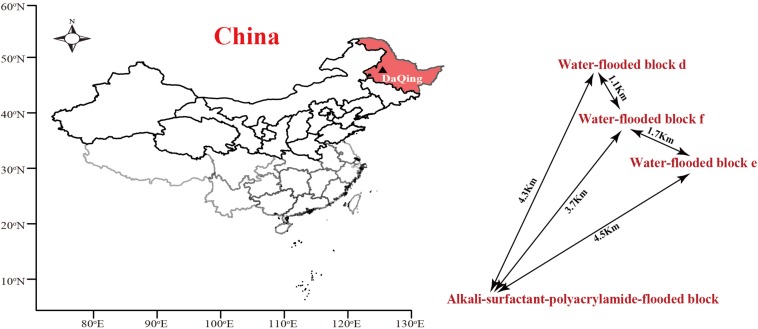
The location of Daqing Oilfield and distribution of the ASP-flooded block and adjacent water-flooded block d, e, and f. The map was drawn using R (i386 3.1.2) with open source packages “maps,” “mapdata,” and “maptools.”

The ASP-flooded block and its adjacent water-flooded block are located in Daqing Oil Field, Northeast China. The temperature of oil-bearing strata of the reservoir is approximate 45°C. The distances between the sampled ASP-flooded block and each water-flooded block (d, e, f) range from one to several kilometers (Figure 1). The average inter-well spacing of the block is about 250 m. The oil reservoir has been exploited by water-flooding for decades, with an average water content of the produced liquid of above 90%. ASP-flooding was started from March 2013, and finished August 2018. The pH of the production brines obtained from the ASP-flooded and water-flooded blocks were approximate 11.5 and 6.5, respectively, and the salinities of the formation brines were about 13196 mg/L and 8678 mg/L, respectively.

Four oil-water samples were collected from oil production wells of the ASP-flooded block and labeled as ASP1, ASP2, ASP3, and ASP4. Ten oil-water samples were collected from oil production wells of the adjacent water-flooded block d, e, and f, and labeled as d8, d9, d10, d11, e5, e6, e7, f5, f6, and f7. The samples were collected through sampling valves at the wellheads of the production wells, and were stored in 10 L sterilized plastic buckets, which were then sealed with screw caps to avoid contamination and oxygen intrusion. All the samples were transported immediately to laboratory for analysis.

### DNA Extraction

Approximate 4–5 L mixed oil/water liquid from the ASP-flooded production wells and 2–3 L from the water-flooded production wells were centrifuged at 4°C and 12000 × *g* for 20 min in a high-speed centrifuge (Beckman, United States) to pellet microbial cells. Total genomic DNA was extracted by a bead shaker treatment combined with AxyPrep^TM^ Genomic DNA Miniprep Kit (United States) as previously described (Gao et al., 2016).

### 16S rRNA Gene Sequencing and Statistical Analysis

Universal prokaryotic primers 515f (5′-GTG CCA GCM GCC GCG GTA A-3′) and 907r (5′-CCG TCA ATT CMT TTR AGT TT-3′) (Weisburg et al., 1991), 524f (5′-TGY CAG CCG CCG CGG TAA-3′) and Arch958r (5′-YCC GGC GTT GAV TCC AAT T-3′) were used to amplify bacterial and archaeal 16S rRNA genes, respectively. Polymerase chain reaction amplicons in triplicate of the same sample were mixed to avoid bias. Amplicons sequencing was performed on Illumina MiSeq PE300 platform in Majorbiogroup, Shanghai, China. The data were analyzed on the free online platform of Majorbio I-Sanger Cloud Platform^1^. Pairs of reads were merged using FLASH (Magoc and Salzberg, 2011), and then were demultiplexed using QIIME2 (Bolyen et al., 2018). To reduce sequencing deviation, 25332 bacterial and 29421 archaeal sequences were drawn out at random for each sample for bacterial and archaeal community analysis. Uparse was employed to select OTUs at 97% similarity (Edgar, 2013). The representative sequence sets were aligned and given a taxonomic classification using RDP pipeline at an 80% confidence level (Wang et al., 2007; Cole et al., 2014). The differences of microbial alpha diversity indices of the bacterial and archaeal communities were determined by One Way Analysis of Variance (one-way ANOVA) and Student-Newmnan-Keuls test. Principal Coordinates Analysis (PCoA) based on Weighted and Unweighted unifrac metric and hierarchy clustering based on Bray-Curtis distances of the OTUs were used to visualize the differential distribution of the microbial communities inhabiting the ASP-flooded and water-flooded blocks. ANOSIM and Adonis analysis based on Bray-Curtis distances of the OTUs were performed to investigate whether significant differences exist between the microbial communities. Wilcoxon rank-sum test was performed to determine the microbial populations with statistical difference between the ASP-flooded and water-flooded blocks. Circos^2^ was used to explore the relationships between the dominant populations and reservoir blocks (Krzywinski et al., 2009).

### Nutrients Stimulation in Microcosms

Dissolved electron acceptors, in particular oxygen and nitrate, are naturally absent in deep oil reservoirs unless anthropogenic introduction. To reveal the responses of the microbial communities in the ASP-flooded block to nutrients, 0.2 g corn steep powder, 0.15 g (NH_4_)_2_HPO_4_, and 0.2 g NaNO_3_ were added to 300 mL serum bottle containing 100 mL produced brines and 0.5 g crude oil. The pH of the microcosms was adjusted to 11. The bottles were then sealed with rubber stoppers to provide a facultative anaerobic environment. Cultivation was accomplished at 45°C for 15 days. The pH of the enrichment culture was measured using pH test strips. Surface tension of the enrichment culture was measured using a digital tension-meter (POWEREACH JK99B, China) at room temperature. The number of cultivable microorganisms were measured based on colony forming unit counts: aliquots of 10^3^ to 10^5^ dilutions of the above enrichment cultures were plated onto agar medium plates prepared with produced brines of the ASP-flooded block, then, were incubated at 45°C in an aerobic condition till colony formation. The medium consists of 2 g/L corn steep powder, 1.5 g/L (NH_4_)_2_HPO_4_, 2 g/L NaNO_3_, and 5 g/L crude oil, with pH of 11. At the same time, microbial cells were collected from 20 mL of the enrichment cultures by centrifugation at 4°C and 12000 × *g* for 20 min. Total genomic DNA extraction and 16S rRNA gene sequencing were performed as described above.

### 16S rRNA Gene Identification of Strain

Microorganisms were isolated from the above agar medium plates. The genomic DNA of the isolated strains was extracted with AxyPrep^TM^ Genomic DNA Miniprep Kit (Axygen, United States). Microbial 16S rRNA gene was amplified using universal primer set 27f (5′-AGA GTT TGA TCT GGC TCA G-3′) and 1492r (5′-TAC GGT TAC CTT GTT ACGACTT-3′) (Winker and Woese, 1991). The amplicons were sequenced and compared with sequences deposited in GenBank database. Phylogenetic tree was constructed using MEGA 4 based on Neighbor-Joining method (Saitou and Nei, 1987; Tamura et al., 2007).

### Sequences Accessibility

The raw reads from Illumina MiSeq sequencing were deposited in Sequence Read Archive (SRA) at National Center for Biotechnology Information^3^^,4^. The 16S rRNA gene sequences of the isolated strains were deposited in Gene Bank databases under accession numbers: S1, MK156726; S2, MK156727; S3, MK156732; S4, MK156733; S5, MK156728; S6, MK156729; S7, MK156730; S8, MK156731.

## Results

### Microbial α-Diversity Indices of the ASP-Flooded Block and Water-Flooded Block

**FIGURE 2:**
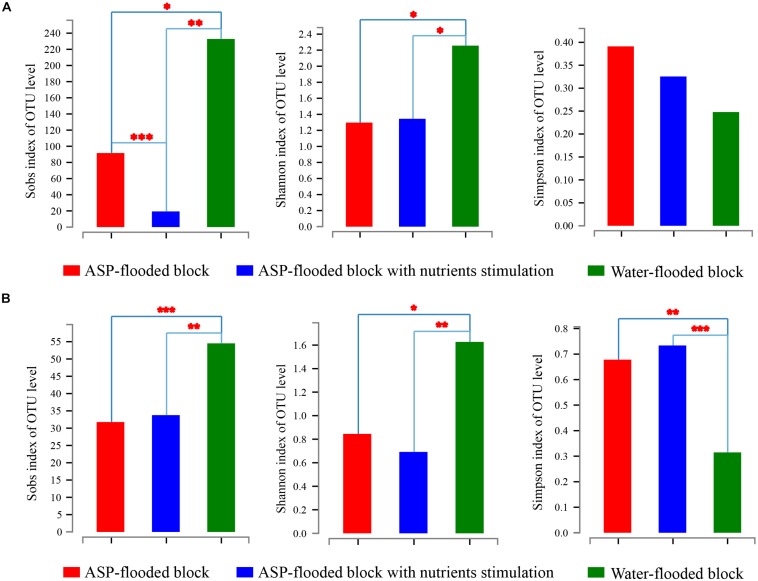
The Sobs, Shannon, and Simpson indices of bacterial **(A)** and archaeal **(B)** communities in the ASP-flooded block and adjacent water-flooded block. The indices were calculated at 3% genetic divergence level of the OTUs. One Way Analysis of Variance (one-way ANOVA) with Student-Newmnan-Keuls test was employed to reveal the statistical significance between the ASP-flooded block and adjacent water-flooded block. ^∗^ represent *P* < 0.05, ^∗∗^ represent *P* < 0.01, ^∗∗∗^ represent *P* < 0.001.

Community coverage was calculated by Good’s coverage equation (Good, 1953). Both the bacterial and archaeal sequencing coverages reached 99.73–99.86% and 99.97–99.98%, respectively, and thus could represent the bacterial and archaeal communities in each sample. The Sobs, Shannon, and Simpson indices of the bacterial and archaeal communities were calculated at 3% genetic divergence level of the bacterial and archaeal OTUs, respectively (Table 1). Compared with the bacterial and archaeal communities of the water-flooded block, lower Sobs (91:232, 34:55) and Shannon indices (1.296:2.256, 0.845:1.627), and higher Simpson indices (0.391:0.248, 0.678:0.315) were observed in the ASP-flooded block (Table 1 and Figure 2). The results indicated that ASP-flooding decreased microbial diversity, and a number of bacterial and archaeal populations still survived in the ASP-flooded reservoir.

**TABLE 1:** The sequencing information and alpha diversity indices of the bacterial and archaeal communities inhabiting the ASP-flooded block and adjacent water-flooded block.

	**α-diversity indices**	**ASP-flooded block^a^**	**ASP-flooded block with nutrients stimulation^b^**	**Water-flooded block^c^**	***P*-value (a-b)**	***P*-value (a-c)**	***P*-value (b-c)**
**Bacterial community**	Reads	25332	25332	25332	–	–	–
	Coverage	0.9986	0.9997	0.9973	–	–	–
	Sobs	91.5 ± 3.1	19.25 ± 7.27	232.7 ± 96.85	<0.001	0.015	<0.001
	Shannon	1.296 ± 0.123	1.341 ± 0.06	2.256 ± 0.801	0.511	0.038	0.046
	Simpson	0.391 ± 0.06	0.325 ± 0.016	0.248 ± 0.164	0.086	0.122	0.376
**Archaeal community**	Reads	29421	29421	29421	–	–	–
	Coverage	0.9998	0.9998	0.9997	–	–	–
	Sobs	31.75 ± 6.5	33.75 ± 5.68	54.5 ± 9.42	0.659	<0.001	0.0015
	Shannon	0.845 ± 0.449	0.691 ± 0.2	1.627 ± 0.446	0.556	0.012	0.0018
	Simpson	0.678 ± 0.193	0.734 ± 0.08	0.315 ± 0.124	0.613	0.0011	<0.001

*The differences of the α diversity indices among the microbial communities were compared via One Way Analysis of Variance with Student-Newmnan-Keuls test. The letter a, b, and c represents ASP-flooded block, ASP with nutrients stimulation, and Water-flooded block, respectively.*

### Composition of Bacterial Communities in the ASP-Flooded Block and Water-Flooded Block

**FIGURE 3:**
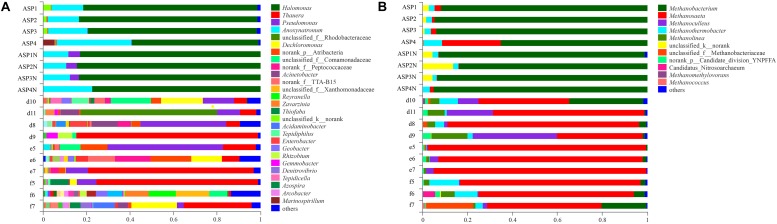
Bacterial **(A)** and archaeal **(B)** community compositions at the genus level in the ASP-flooded production wells and adjacent water-flooded production wells.

**FIGURE 4:**
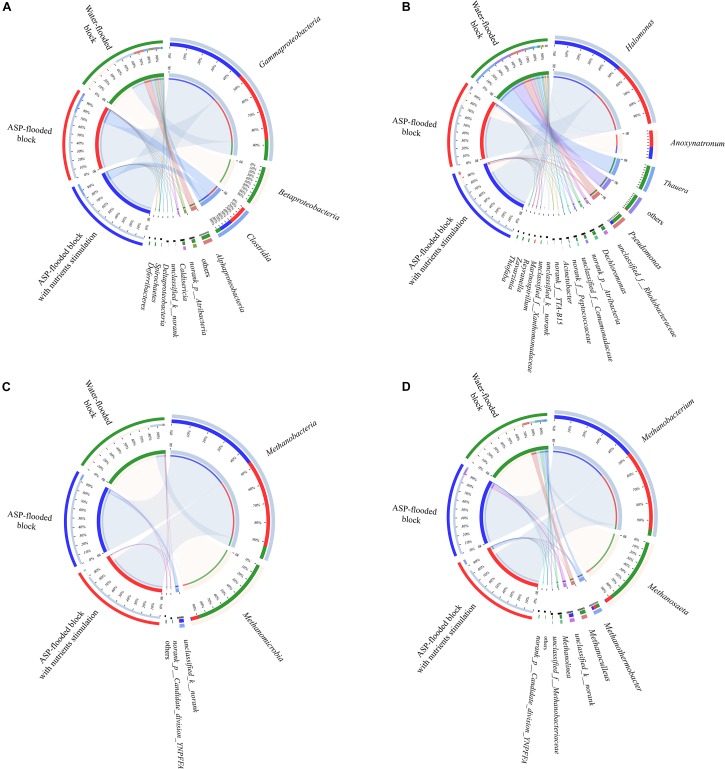
Circos plots revealed the distribution of dominant bacterial **(A,B)** and archaeal **(C,D)** populations in the ASP-flooded block and adjacent water-flooded block. The percentages on the left semicircle represented the relative abundances of the dominant taxa in each site.

**FIGURE 5:**
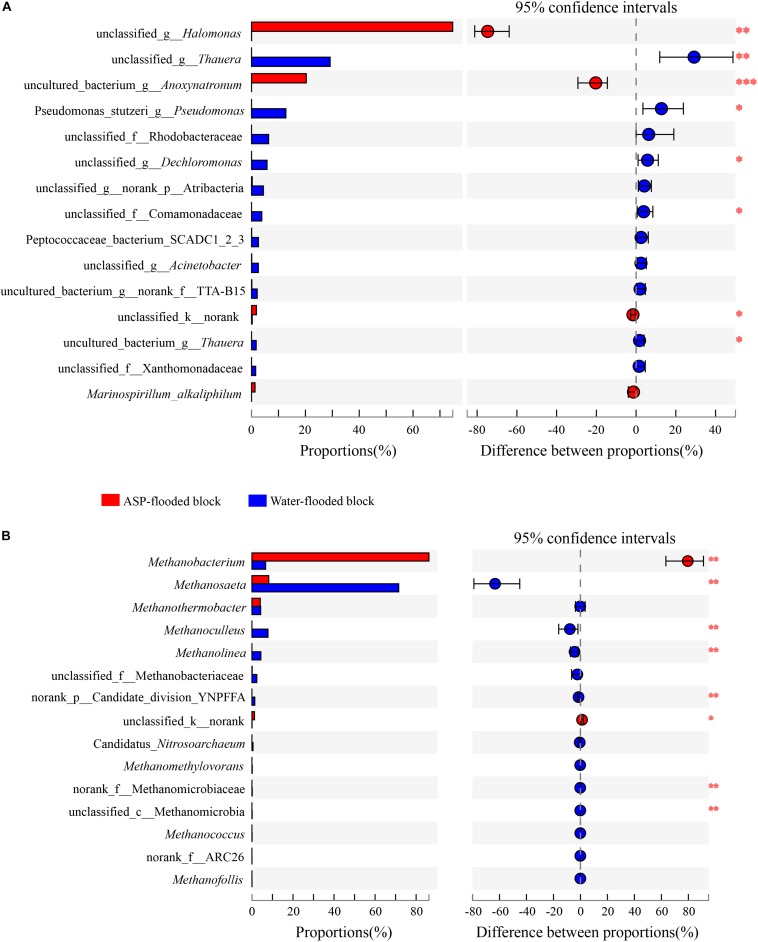
Wilcoxon rank-sum test revealed the bacterial **(A)** and archaeal **(B)** populations with significant differences in the relative abundance between the ASP-flooded block and adjacent water-flooded block. ^∗^ represent *P* < 0.05, ^∗∗^ represent *P* < 0.01, ^∗∗∗^ represent *P* < 0.001.

Compared with the bacterial communities that colonized the water-flooded production wells, the communities in the ASP-flooded production wells showed higher similarity (Figure 3A). As shown in Figure 4A, Gammaproteobacteria and Clostridia were most frequently detected in the ASP-flooded block, Betaproteobacteria and Alphaproteobacteria predominated in the adjacent water-flooded block. At the genus level, *Halomonas* and *Anoxynatronum* were dominated populations of the ASP-flooded production wells Figure 4B, and accounted for 58.39–82.12% and 14.46–18.15% of the microbial communities, respectively. Of special note is that the relative abundances of the above populations were less than 0.05% in the adjacent water-flooded production wells, where *Thauera*, *Pseudomonas*, *Rhodobacteraceae*, and *Acinetobacter* were dominant populations. Wilcoxon rank-sum test indicated that *Pseudomonas*, *Thauera*, *Halomonas*, *Anoxynatronum* and *Marinospirillum alkaliphilum* were the dominant populations with significantly differential relative abundances between the water-flooded block and ASP-flooded block (Figure 5A).

A total of 183 and 1081 bacterial OTUs were detected in the ASP-flooded and water-flooded production wells, respectively. Among them, 139 OTUs were detected in both the two blocks. The relative abundances of the shared OTUs were obvious differences between the ASP-flooded and water-flooded production wells: OTU4528 that belonged to *Halomonas* accounted for 14.52–40.79% in the ASP-flooded production wells, whereas it only accounted for 0–0.15% in the water-flooded production wells; OTU2753 that belonged to *Thauera* and OTU4537 that belonged to *Pseudomonas* predominated the water-flooded production wells, and were rare in the ASP-flooded production wells. There were 44 OTUs that existed in the ASP-flooded production wells and were not detected in the water-flooded production wells. Among them, OTU4537 belonged to *Anoxynatronum* and OTU4533 belonged to *Halomonas* accounted for 14.45–34.21% and 3.47–7.52% of the microbial communities, respectively.

### Composition of Archaeal Communities in the ASP-Flooded Block and Water-Flooded Block

The 97.28–99.93% archaeal sequences identified in the ASP-flooded production wells and 91.59–99.67% archaeal sequences identified in the water-flooded production wells belonged to Methanomicrobia and Methanobacteria. However, Methanobacteria was most frequently detected in the ASP-flooded production wells, accounting for 73.59–97.18% of the archaeal communities, and Methanomicrobia predominated in the adjacent water-flooded production wells (Figure 4C), accounting for 56.26–99.11%. At the genus level, *Methanobacterium* was the dominated population in the ASP-flooded production wells (Figure 4D), accounting for 65.01–93.86% of the archaeal communities (Figure 3B). In the water-flooded production wells, the relative abundances of *Methanosaeta* ranged from 36.72 to 96.90% (Figure 3B). Wilcoxon rank-sum test showed that the relative abundances of *Methanobacterium*, *Methanosaeta*, *Methanoculleus*, and *Methanolinea* had significant differences between the water-flooded block and ASP-flooded block (Figure 5B).

A total of 49 and 119 archaeal OTUs were detected in the ASP-flooded and water-flooded production wells, respectively, with 36 OTUs were both detected. Similar with the bacterial communities, the shared archaeal OTUs between the ASP-flooded block and water-flooded block showed obviously different relative abundances; for instance, OTU90 that belonged to *Methanobacterium* accounted for 60.38–89.32% in the ASP-flooded production wells, whereas it only accounted for 0.006–0.42% in the water-flooded production wells. There were 13 OTUs that existed in the ASP-flooded block and were not detected in the water-flooded block. Furthermore, all of the 13 OTUs belonged to unclassified archaea, and accounted for 0.05–2.71% of the archaeal communities.

### Distribution of the Microbial Communities in the ASP-Flooded Block and Water-Flooded Block

**FIGURE 6:**
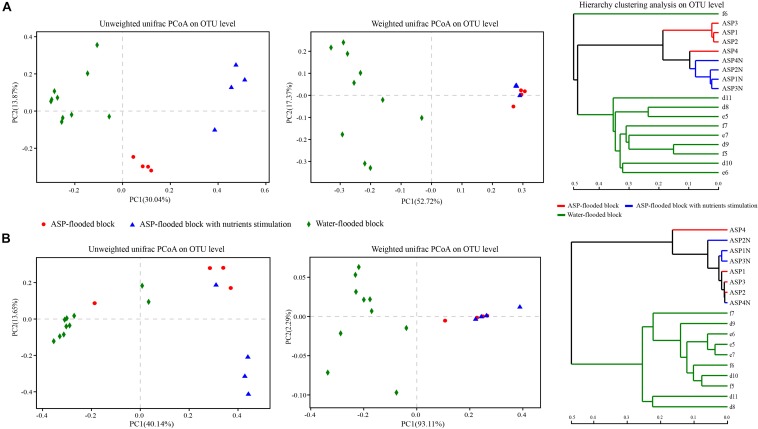
Weighted and Unweighted unifrac PCoA and hierarchy clustering analysis of the bacterial **(A)** and archaeal **(B)** communities in the ASP-flooded block and adjacent water-flooded block. Principal Coordinates Analysis (PCoA) was performed based on Weighted and Unweighted unifrac metric of the OTUs, and hierarchy clustering was performed based on Bray-Curtis distances of the OTUs.

Weighted and Unweighted unifrac PCoA and hierarchy clustering analysis showed that both the bacterial and archaeal communities from the ASP-flooded production wells clustered together, and were far apart from those of the adjacent water-flooded production wells (Figure 6). ANOSIM and Adonis analysis based on Bray-Curtis distances of the OTUs indicated that the ASP-flooded block harbor significantly differential bacterial communities (*R* = 0.99, *P* < 0.01, and *F*_1, 12_ = 6.77, *R* = 0.60, *P* < 0.01, respectively) and archaeal communities (*R* = 1, *P* < 0.01, and *F*_1, 12_ = 32.30, *R* = 0.85, *P* < 0.01, respectively) compared with those of the adjacent water-flooded block.

### Responses of the Microcosms From the ASP-Flooded Block to Nutrients

After nutrients stimulation, the pH of the brines of the microcosms decreased from 11 to 10, and the number of cultivable microorganisms increased from 10^3^/mL to 10^7^/mL. However, the changes of the surface tensions of the brines were not obvious. Oil emulsification or dispersion was also not obviously observed. Hierarchy clustering based on Bray-Curtis distances of the OTUs showed the differentiation of the bacterial and archaeal communities in the produced brines with or without nutrients stimulation (Figure 6). In addition, both the bacterial and archaeal communities of the produced brines with or without nutrients stimulation clustered together in the Weighted unifrac PCoA plots, and were far apart in the Unweighted unifrac PCoA plots (Figure 6). The phenomena indicated that both the bacterial and archaeal communities in the produced brines changed before and after nutrients stimulation, yet there were no significant changes in the relative abundances of dominant populations. ANOSIM analysis indicated that the differences between the bacterial and archaeal communities from the produced brines and nutrients stimulated produced brines were not significant (*R* = 0.531, *P* = 0.083; *R* = 0.125, *P* = 0.085, respectively).

**FIGURE 7:**
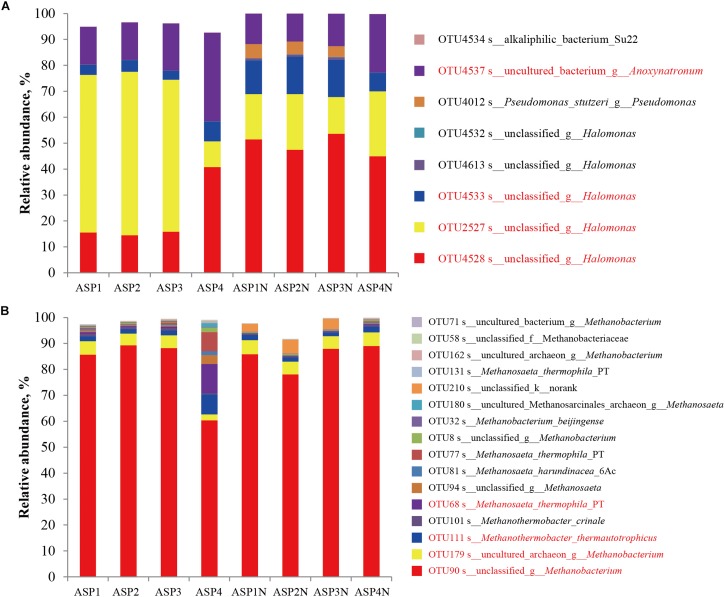
The distribution of dominant bacterial **(A)** and archaeal **(B)** populations in the brines collected from ASP-flooded block with or without nutrients stimulation.

The Sobs indices of the bacterial communities in the nutrients stimulated produced brines significantly decreased (Table 1 and Figure 2) and the relative abundances of some species increased (Figure 7). Among them, OTU4528 that belonged to *Halomonas* increased from 14.52–40.79% to 45.01–53.68% after nutrients stimulation, and OTU4533 that belonged to *Halomonas* increased from 3.47–7.52% to 7.07–14.40% (Figure 7A). In addition, OTU4012 affiliated with *Pseudomonas stutzeri* that accounts for less than 0.01% in the production wells also obviously increased after nutrients stimulation, with the relative abundances reached to 4.15–5.40% (Figure 7A). For the archaeal communities, the α indices of the produced brines did not obviously changed before and after nutrients stimulation (Table 1 and Figure 2). Except for ASP4, there were no obvious changes of the relative abundances of dominant OTUs (Figure 7B).

### Strains Isolated From the Nutrients Stimulated Brines

**FIGURE 8:**
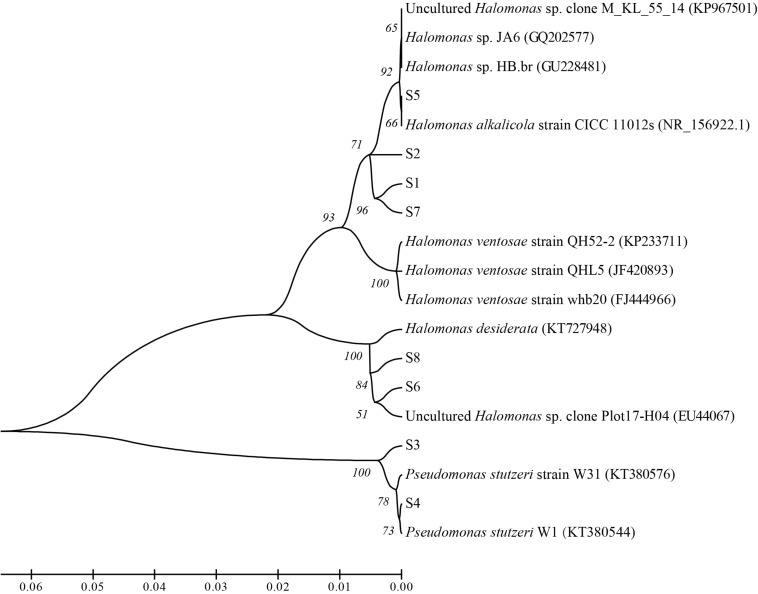
Phylogenetic relationships between the isolated strains from the nutrients stimulated brines from the ASP-flooded block.

From the nutrients stimulated produced brines, 8 strains with differential colonial morphology were isolated. Phylogenetic analysis based on 16S rRNA gene sequences showed that 6 strains belonged to *Halomonas* sp. and 2 strains belonged to *Pseudomonas stutzeri*. Among them, strain S1, S2, S5, and S7 showed 98% similarity of 16S rRNA genes with those of *Halomonas alkalicola*, strain S6 and S8 showed 98% similarity with *Halomonas desiderata* gene, and strain S3 and S4 showed 99% similarity with *Pseudomonas stutzeri* gene (Figure 8).

## Discussion

This study investigated the influence of ASP-flooding on composition of microbial communities inhabiting an oil reservoir exploited by water-flooding for decades by comparing with those of the adjacent water-flooded production wells. The results showed that the ASP-flooded production wells had lower microbial diversity. This is in accordance with the results of an our previous investigation on microbial communities in diverse oil reservoirs: both the bacterial and archaeal diversity were significantly influenced by extreme reservoir environments, such as steam-soaking, high temperature, high pressure and hypersalinity (Gao et al., 2016). Here, the injected alkali, surfactants, and hydrolyzed polyacrylamide have influenced the microbial communities in the ASP-flooded block. Particularly, the injected alkali created a strong alkaline environment, with pH of the formation brines up to 11.5, which is much higher than those of most water-flooding oil reservoirs, with pH of the formation brines ranging from 6 to 8 (Wang et al., 2012; Lenchi et al., 2013; Gao et al., 2016). The pH exceeds the survival limits of most microbial populations. As expected, fewer OTUs, lower Shannon indices, and higher Simpson indices were remarkably observed in microbial communities of the ASP-flooded production wells when were compared with those of the water-flooded production wells. Furthermore, alkali-tolerating populations *Halomonas* and *Anoxynatronum* dominated the ASP-flooded production wells, yet were less than 0.05% in the bacterial communities of the adjacent water-flooded production wells. The phenomena indicated that many microbial populations inhabiting water-flooded block disappeared in ASP-flooding process because of the harsh selection pressure of extreme environment, and fewer microbial populations that well adapted to or tolerant of the extreme environment survived.

Niche differences, in particular temperature (Piceno et al., 2014; Sharp et al., 2014; Li et al., 2017), salinity (Auguet et al., 2010), pH (Lauber et al., 2009; Kuang et al., 2013) and substrates (Hallmann et al., 2008; N’Guessan et al., 2010; Lai et al., 2014; Conlette et al., 2016; Liu et al., 2018), are considered the major determinants of microbial community assembly. For oil reservoirs, geographical isolation and water cut of production wells, and low permeability of oil-bearing strata will also exert significant influences on microbial community composition (Ren et al., 2015; Gao et al., 2016; Song et al., 2017). In addition, stochastic process is considered another important factor that influences microbial community assembly (Stegen et al., 2012; Guo et al., 2018). Actually, many researches have reported that microbial communities were highly heterogeneous even in the adjacent production wells that were flooded by same injected water (Ren et al., 2015; Gao et al., 2016; Song et al., 2017). Similarly, *Thauera*, *Pseudomonas*, *Rhodobacteraceae*, and *Acinetobacter* were dominant populations in the water-flooded block, yet their relative abundances showed obvious differences in the production wells. Thus, it appears that stochastic process plays an important role in determining the microbial communities in the water-flooded production wells. In contrast, microbial communities in the ASP-flooded oil production wells showed a higher similarity in community composition, with *Halomonas* (58.39–82.12%) and *Anoxynatronum* (14.46–18.15%) accounting for 92.64–96.59% of the communities. Apparently, the alkaline environment served as a major selective force rather than stochastic process in the ASP-flooded block.

*Halomonas* and *Anoxynatronum* dominated the ASP-flooded block. *Halomonas* species have been detected in diverse saline environments, including estuaries, ocean, saline lakes. These microorganisms belong to halophilic or salt-tolerating proteobacteria (Vreeland, 2015). Species of *Anoxynatronum* had been reported as anaerobic alkaliphilic bacteria that can grow in pH 7.4–11.0 with optimum pH of 9.6 (Ryzhmanova et al., 2017). As shown in Table 2, *Halomonas* and *Anoxynatronum* have also been detected in some oil reservoirs, and were rare (Gao et al., 2016). *Pseudomonas* and *Thauera* were most frequently detected populations in water-flooded reservoirs (Wang et al., 2012; Lenchi et al., 2013; Vigneron et al., 2017). The phenomena appear to indicate that *Halomonas* and *Anoxynatronum* have no competitive edges when coexist with *Pseudomonas* and *Thauera* in water-flooded reservoirs.

**TABLE 2:** Distribution of the dominant bacterial and archaeal populations in the ASP-flooded reservoir and the other Chinese oil reservoirs.

Oilfield	Block	°C	*Anoxynatronum*	*Halomonas*	*Pseudomonas*	*Thauera*	*Methanobacterium*	*Methanosaeta*	*Methanothermobacter*
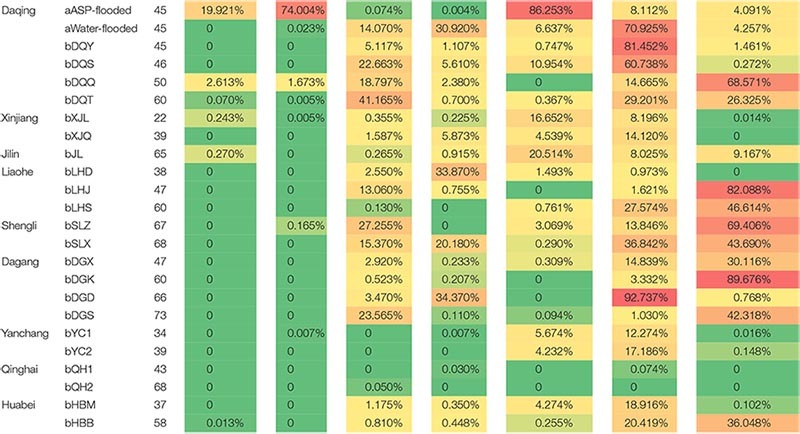									

*Except for YC2, all of the above blocks had been water-flooded at some point in the past. Steam soaking technique had been performed in the LHJ reservoir block. ^*a*^16S rRNA gene sequencing was performed on an Illumina MiSeq platform; ^*b*^16S rRNA gene sequencing was performed on a Roche Genome Sequencer GS FLX + platform. The color gradient ranging from green to red represents the increase of relative abundance.*

Methylotrophic and hydrogenotrophic *Methanobacterium* (Liu and Whitman, 2008) was the dominant archaeal population in the ASP-flooded block. Thus, it is not difficult to speculate *Methanobacterium* is of unique salt tolerating ability. A small amount of *Methanosaeta* that only uses acetate in methane production (Liu and Whitman, 2008) was also detected. Although *Methanobacterium* exists widely in water-flooded oil reservoirs, its relative abundances are far less than *Methanosaeta* and *Methanothermobacter* (Table 2).

The AMP-flooding experiment (Wang et al., 2019) prompts us to concern the EOR potential of microorganisms inhabiting ASP-flooded reservoirs. Microbial enhanced oil recovery (MEOR) depends on microbial growth and produced metabolites, such as biosurfactants, acid, biogases, and biopolymer. To reveal the above concerns, nutrients stimulation of the microbial communities from the ASP-flooded block was performed in microcosms. The results indicated that the microbial communities showed significant responses to nutrients addition: the number of cultivable microorganisms increased from 10^3^/mL to 10^7^/mL, the relative abundances of some OTUs that belonged to *Halomonas* and *Pseudomonas* obviously increased. Oil emulsification, oil dispersion and/or decrease of surface tension are the main ways for MEOR (Youssef et al., 2009; Voordouw, 2011). However, oil emulsification or dispersion was not obviously observed in nutrients stimulation process, and the surface tensions of the brines did not drastically decrease. The phenomena indicated that the extreme environment of the ASP-flooded block restricted microbial growth and metabolisms to some extent. Therefore, it is hard to stimulate these microorganisms to enhance oil recovery by nutrients injection through injection wells in ASP-flooded reservoirs except that new communities form in subsequently water-flooding process.

In view of the isolation and culture methods, 6 alkali-tolerating strains with 98% similarity of 16S rRNA genes to *Halomonas alkalicola*, *Halomonas ventosae* and *Halomonas desiderata* genes, and 2 alkali-tolerating strains showing 99% similarity with *Pseudomonas stutzeri* gene were isolated. *was* reported as an alkaliphilic and halotolerant bacterium that can propagate at pH 12.5 (Zhai et al., 2018). *Halomonas ventosae* was reported as a moderately halophilic, denitrifying, exopolysaccharide-producing bacterium (Martínez-Cánovas et al., 2004). *Halomonas desiderata* was reported as an alkaliphilic, halotolerant and denitrifying bacterium (Berendes et al., 1996). These data give an indication of microbial adaption to the harsh alkaline condition, yet are not able to reflect the activity of microbial metabolisms in ASP-flooded block.

## Conclusion

This study revealed the characteristics of microbial communities in an ASP-flooded block by comparing the microbial communities with those of the adjacent water-flooded block, and the responses of microbial communities in the ASP-flooded block to nutrients in microcosms. The results indicated that the extreme environment of the ASP-flooded reservoir significantly decreased microbial diversity, leading alkali-tolerating *Halomonas*, *Anoxynatronum*, and *Methanobacterium* predominated. The microbial communities in the ASP-flooded reservoir could be stimulated by provided nutrients, yet the harsh alkaline condition restricted microbial growth and metabolisms. The results also provided insights about the application potential for MEOR in ASP-flooded reservoir: it is hard to stimulate these microorganisms to enhance oil recovery by nutrients injection through injection wells except that new communities form in subsequently water-flooding process.

## Data Availability Statement

All datasets generated for this study are included in the manuscript/supplementary files.

## Author Contributions

PG and TM designed the project. PG, YL, LT, and FG conducted the experiments and wrote the manuscript. All authors critically reviewed the manuscript.

## Conflict of Interest

LT was employed by PetroChina Daqing Oilfield Limited Company. The remaining authors declare that the research was conducted in the absence of any commercial or financial relationships that could be construed as a potential conflict of interest.

## Footnotes

1 www.i-sanger.com

2 http://circos.ca/

3 https://www.ncbi.nlm.nih.gov/bioproject/PRJNA505232

4 https://www.ncbi.nlm.nih.gov/bioproject/PRJNA489604
